# Volumetric Biomarkers of Visual Outcome after Surgical Repair in Lamellar Macular Holes

**DOI:** 10.3390/jpm14070755

**Published:** 2024-07-16

**Authors:** Myrta Lippera, George Moussa, Tsveta Ivanova, Mariantonia Ferrara, Karina Spiess, Naseer Ally, Kirti Jasani, Felipe Dhawahir-Scala, Niall Patton, Assad Jalil

**Affiliations:** 1 Manchester Royal Eye Hospital, Manchester University Hospitals NHS Foundation Trust, Manchester M13 9WL, UKkarinaspiess@gmx.net (K.S.); kirti.jasani@mft.nhs.uk (K.J.);; 2 School of Medicine, University of Malaga, 29071 Malaga, Spain; 3 Department of Medical and Surgical Specialties, Radiological Sciences and Public Health, University of Brescia, 25123 Brescia, Italy; 4 Eye Unit, ASST Spedali Civili di Brescia, Piazzale Spedali Civili, 25123 Brescia, Italy; 5 Division of Ophthalmology, Department of Neurosciences, School of Clinical Medicine, Faculty of Health Sciences, University of the Witwatersrand, 7 York Road, Parktown, Johannesburg 2193, South Africa

**Keywords:** lamellar macular hole (LMH), optical coherence tomography (OCT), predictive prognostic factors, biomarkers, retinal layer segmentation, retinal volume, volumetric analysis

## Abstract

Background: We investigate novel OCT parameters, based on the volumetric analysis of lamellar macular holes (LMHs), as prognostic indicators for visual outcomes after surgery. Methods: LMHs were divided into degenerative LMHs (D-LMHs) and ERM-foveoschisis (ERM-FS). Pre-operative clinical, OCT linear and volumetric parameters were collected. Volumes were obtained using the OCT automatic segmentation, such as central retinal volume (CRV) and outer nuclear layer (ONL) volume, or using a novel method to calculate volumes of specific LMH entities like epiretinal proliferation (ERP), foveal cavity (FC) in D-LMH and schitic volume (SV) in ERM-FS. Univariate and multivariate linear regression analysis evaluated the factors predictive for post-operative best-corrected visual acuity (BCVA). Results: We included 31 eyes of 31 patients (14 D-LMH,17 ERM-FS). A pre-operative BCVA ≤ 0.48 logMAR was a predictor for achieving ≤0.30 logMAR at final follow-up. A lower pre-operative BCVA (*p* = 0.008) and the presence of ERP (*p* = 0.002) were associated with worse visual outcomes post-surgery. Moreover, novel pre-operative OCT parameters significantly associated with worse post-operative BCVA, such as increased FC volume (*p* = 0.032) and lower CRV (*p* = 0.034) in the D-LMH subtype and lower CRV (*p* < 0.001) and ERP volume (*p* < 0.001), higher SV (*p* < 0.001) and foveal ONL volume (*p* < 0.001) in the ERM-FS subtype. Conclusions: Novel volumetric OCT parameters can be prognostic indicators of visual outcome following surgery in LMHs.

## 1. Introduction

Lamellar macular hole (LMH) is morphologically characterized by the presence of a partial defect in the inner foveal layers, not extending to the entire retina, with irregular foveal contour [1,2,3]. With the advent of optical coherence tomography (OCT), different classifications and subtypes of LMH have been proposed [1,2,3,4]. More recently, an OCT-based consensus renamed two clinical subtypes: “epiretinal membrane-foveoschisis” (ERM-FS), characterized by the presence of a contractile ERM and foveoschisis at the level of Henle fiber layer (HFL) and a (degenerative) LMH, characterized by a foveal cavity with undermined edges and signs evoking loss of foveal tissue [2]. The pathophysiology of LMH is still largely unknown [2,5]; however, two main mechanisms have been described: (i) a contractile membrane causing traction in ERM-FS and (ii) loss of retinal tissue in D-LMH [2]. Both mechanisms produce asymmetric three-dimensional (3D) changes on the macula.

LMH is a slowly progressive condition that can impair visual acuity (VA) in a subset of patients [6]. Although pars plana vitrectomy (PPV) and internal limiting membrane (ILM) peeling is the established surgical approach for LMH, there is no general consensus regarding quantitative parameters that can help decide on the timing of surgery or predict the visual outcome [7,8,9]. It has been highlighted that LMH is an asymmetric, 3D disease of the macula, better defined by volumetric analysis rather than linear measurements [6]. Most previous studies on LMH, were limited by the analysis of measurements from linear OCT macular scans, which do not provide adequate information on retinal tissue remodeling [6,10,11].

The aim of our study was to investigate novel OCT parameters, based on the volumetric analysis of ERM foveoschisis (ERM-FS) and degenerative LMH (D-LMH), as prognostic indicators for post-operative best-corrected visual acuity (BCVA) logMAR after surgery. Additionally, due to the functional importance of 0.30 logMAR BCVA, such as for driving, we investigate the effect of pre-operative BCVA on achieving this threshold postoperatively.

## 2. Materials and Methods

This is a retrospective interventional, single-center case series that adhered to the guidelines of the Declaration of Helsinki. Under UK guidance, retrospective data collection is regarded as an audit for the purpose of service evaluation, and as such ethical approval was not required. Diagnosis and treatment were conducted according to local guidelines with no new or experimental protocols. Clinical records were extracted from an electronic surgical database of consecutive eyes that underwent PPV for LMH repair at Manchester Royal Eye Hospital, UK, from January 2020 to January 2023. Eyes with LMH which had pre-operative macular OCT using the “dense macular volume” scan with Heidelberg Spectralis (Engineering GmbH, Heidelberg, Germany), were included. Exclusion criteria were (1) high myopia (more than 6 diopters); (2) advanced glaucoma; (3) any concomitant retinal disease involving the macula, such as diabetic maculopathy, retinal vein occlusion, age-related macular degeneration; (4) any comorbidity potentially impacting on final functional outcomes, such as uveitis or amblyopia; (5) history of trauma; (6) previous intraocular surgery other than cataract surgery performed more than 6 months before vitrectomy; (7) OCT with poor image quality; and (8) post-operative follow-up (FU) less than 4 weeks.

**Figure 1:**
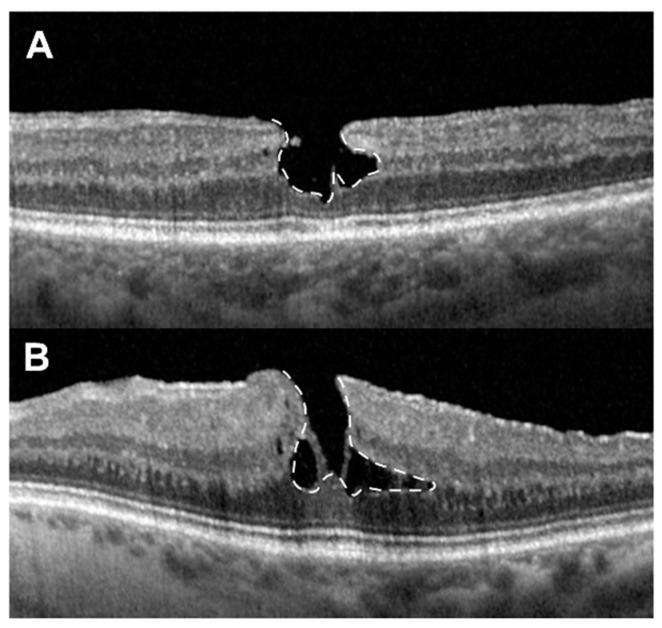
Sub-categories of lamellar macular holes. (**A**) Degenerative lamellar macular hole (D-LMH): defined by the presence of an irregular foveal contour (the normal smooth and regular contour of the fovea is disrupted), a foveal cavity with undermined edges (a cavity or space, highlighted by the white dashed line, is observed in the fovea with edges that appear undermined) and apparent loss of retinal tissue on OCT. (**B**) Epiretinal membrane foveoschisis (ERM-FS): defined by the presence of a contractile ERM (an ERM can be detected on the retinal surface, exerting traction on the retina) with foveoschisis (the schisis within the retinal layers, specifically at the level of Henle’s fiber layer due to the traction from the ERM, is highlighted by the white dashed line). In summary, key differentiating features between D-LMH and ERM-FS include: ERM presence (ERM-FS is defined by the presence of a contractile ERM, while D-LMH might lack this feature); foveal schisis vs. cavity (in ERM-FS, the foveal splitting or schisis is due to traction, often at Henle’s fiber layer, whereas in D-LMH, the foveal cavity with undermined edges is due to degeneration); retinal tissue loss (D-LMH typically shows apparent loss of retinal tissue, whereas ERM-FS might not show this degree of tissue loss but rather separation or schisis within layers); and contour changes (the irregular foveal contour is more pronounced in D-LMH due to degeneration, while in ERM-FS, the changes are primarily due to the tractional effects of the ERM).

We subsequently divided the included eyes in two subtypes, degenerative LMH (D-LMH) and ERM foveoschisis (ERM-FS), based on the OCT-based consensus definition for LMH [2], as defined and shown in Figure 1.

For each patient, the following data were collected:

I. Pre-operative characteristics: baseline characteristics such as age, gender, laterality; clinical characteristics such as lens status, pre-operative BCVA (logMAR), ocular findings at dilated fundoscopy; and pre-operative OCT-based characteristics as detailed below.
II. Surgical characteristics and intraoperative complications.
III. Post-operative characteristics: post-operative BCVA (logMAR) and complications. The ETDRS (Early Treatment Diabetic Retinopathy Study) logMAR (logarithm of the Minimum Angle of Resolution) test was the standardized visual acuity test used to assess a patient’s pre- and post-operative vision.

### 2.1. Surgical Technique

Patients with LMH were considered for surgery if they were symptomatic with significant metamorphopsia and/or worsening of BCVA or documented progression of LMH on the OCT. Small-gauge 25G PPV was performed in all cases. For all phakic patients, cataract surgery was concurrently performed. After vitrectomy, ERM and complete ILM peeling were completed. Air or 20% Sulfur-Hexafluoride (SF6) were used as intraocular tamponade depending on surgeon preference.

### 2.2. OCT Parameters

The OCT examination included a macular volume scan with a 49-line horizontal raster covering an area of 30° by 30° (approximately 125 µm spacing between each scan, depending on the axial length of the eye being examined) centered on the fovea. For each scan, two vitreoretinal experts evaluated the ellipsoid zone (EZ) and external limiting membrane (ELM) and classified them as “normal”, “disrupted” and “absent”. Subsequently, using the caliper tool present, the two vitreoretinal experts measured the minimum retinal thickness (MRT), defined as the smallest distance between the retinal pigment epithelium (RPE) and the inner border of the retinal tissue on the fovea on a line perpendicular to the RPE measured on the linear OCT scan dissecting the fovea; the minimum and maximal linear horizontal diameter of the foveal cavity (FC) in D-LMH and of the foveoschisis in ERM-FS defined as the shortest and longest distance measured horizontally across the foveal cavity or the foveoschisis on the linear OCT scan dissecting the fovea.

**Figure 2:**
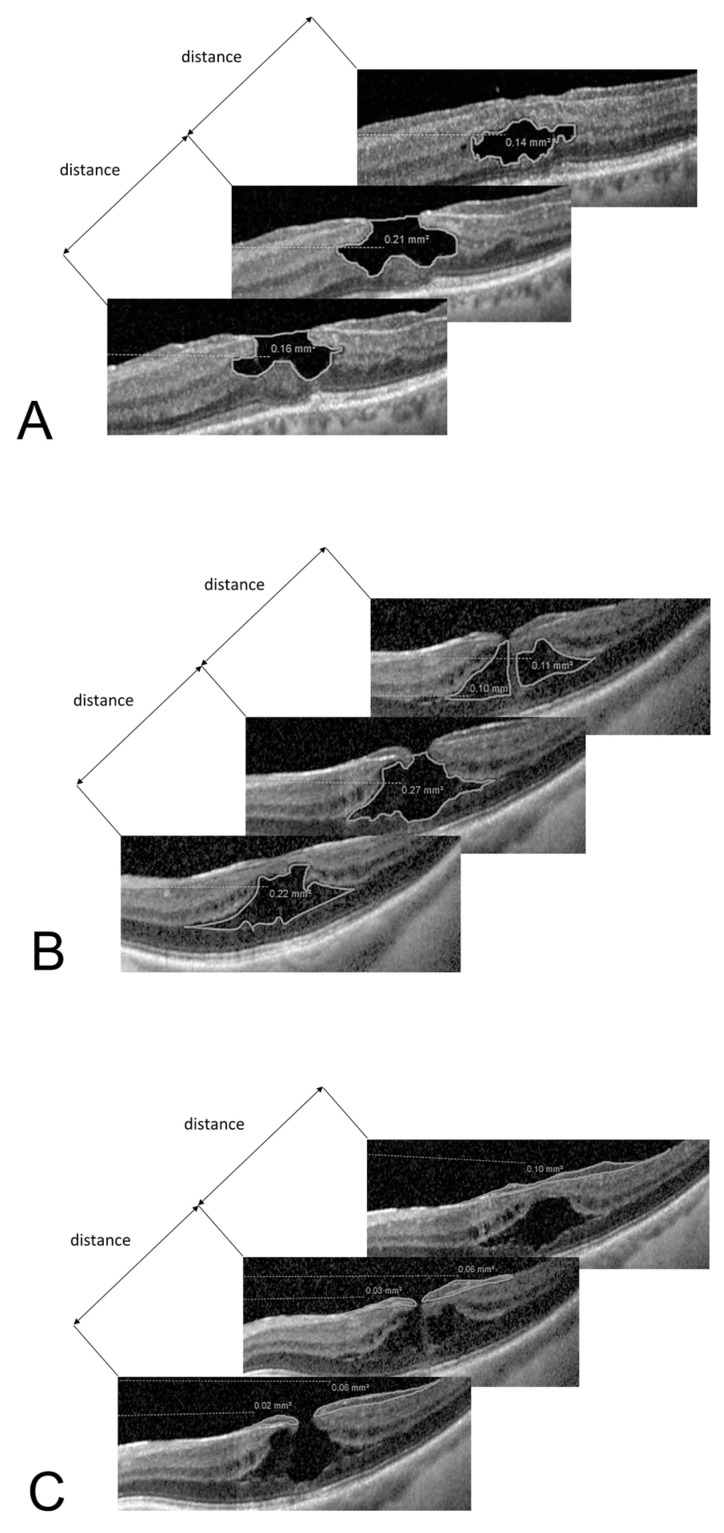
Collection of OCT parameters used to calculate the volumes of specific LMH entities. The areas in yellow are summed and multiplied for the distance between the OCT scans to calculate the estimated volume of a specific LMH entity. (**A**) A specific tool was used to calculate the area of the FC in D-LMH on every single linear OCT scan. Using the formula Volume (mm^3^) = ∑area [mm^2^] × OCT-scan distance [mm], the volume of the FC in D-LMH was calculated. (**B**) A specific tool was used to calculate the area of the SV in ERM-FS on every single linear OCT scan. Using the formula Volume (mm^3^) = ∑area [mm^2^] × OCT-scan distance [mm], the SV in ERM-FS was calculated. (**C**) A specific tool was used to calculate the area of the ERP on every single linear OCT scan. Using the formula Volume (mm^3^) = ∑area [mm^2^] × OCT-scan distance [mm], the volume of the ERP was calculated.

Since the OCT machine does not have the capacity to automatically segment and delineate specific LMH entities like FC in D-LMH, the volume of the schitic cavities described as schitic volume (SV) in ERM-FS, or epiretinal proliferation (ERP), we adopted a novel and manual technique to calculate the volumes of those specific entities, which was validated on healthy and pathological eyes [12]. In brief, we calculate the surface area of the specific entity studied for each linear OCT scan (Figure 2). Finally, the specific volume was calculated by multiplying the sum of the areas by the distance between the horizontal b-scans, using the formula Volume (mm^3^) = ∑area [mm^2^] × OCT-scan distance [mm].

As the last step, after review of the correct OCT automized segmentation of the retinal layers, the following parameters, calculated by the OCT machine, were collected from the “Thickness Map” tablature of the OCT software (V 1.0): the central retinal thickness (CRT), defined as the average linear thickness in the central circle of the 1, 3, 6 ETDRS circle diameters; the central retinal volume (CRV), defined as the volume of the retinal tissue included in the 30° by 30° area scanned; the average ONL thickness, defined as the average thickness of the ONL included in the central circle of the 1, 3, 6 ETDRS circle diameters; and the volume of the ONL within a diameter of 1 mm centered in fovea, defined as the volume of the ONL included in the central circle of the 1, 3, 6 ETDRS circle diameters.

### 2.3. Statistical Analysis

The statistical analysis was executed using IBM SPSS Statistics for Windows, Version 29.0 (IBM Corp, Armonk, NY, USA). Statistical significance was defined as *p* < 0.05. First, continuous variables were estimated for normality using the Shapiro–Wilk test and the mean (standard deviation) was reported for normally distributed variables; otherwise, for skewed data, we reported the median (interquartile range). We performed a paired *t*-test between pre-operative and post-operative continuous variables. Fisher’s exact test was performed to compare nominal variables. To build a model of predictive factors from the initial OCT-based parameters, a multivariable linear regression analysis was conducted with post-operative BCVA (logMAR) as the dependent variable. The lens status, presence and volume of ERP, FC volume in D-LMH and SV in ERM-FS, CRV, foveal ONL volume and pre-operative BCVA (logMAR) were used for the regression model. To demonstrate the different effect of volumetric analyses on D-LMH and ERM-FS, we added interaction terms for foveal ONL volume, ERP volume and CRV as moderators. The 95% confidence interval and *p* values were generated following a 16,000 sample Wild Bootstrap. A Receiver Operating Characteristic (ROC) curve using Area Under Curve (AUC) analysis was performed to report on the sensitivity and specificity of a cut-off threshold based on pre-operative BCVA as a predictor for achieving ≤0.30 logMAR (6/12 Snellen) post-operatively.

## 3. Results

We include 31 eyes of 31 patients with LMH (14 D-LMH, 17 ERM-FS). The mean (standard deviation) age at presentation was 69 [9] years old. Ten patients (32%) were males. Combined vitrectomy with cataract surgery was performed in all phakic patients, which resulted in 21 eyes (68%). No intra or post-operative complications were identified. The baseline and surgical characteristics are found in Table 1.

### 3.1. Pre-Operative Clinical Parameters and Visual Outcomes

The median follow-up period was of 69 days. While in the D-LMH group, BCVA improved from 0.50 (0.24) logMAR pre-operatively to 0.42 (0.18) logMAR at last follow-up (*p* = 0.056); in the ERM-FS group, BCVA improved from 0.49 (0.20) logMAR to 0.32 (0.20) logMAR (*p* = 0.002). Across the whole cohort, mean pre-operative BCVA improved from 0.48 (0.22) logMAR to 0.36 (0.19) logMAR (*p* < 0.001). Pre-operative and post-operative BCVA had a significant correlation (Supplementary Materials, Figure S1). Regarding the potential confounding effect of cataract surgery on visual outcomes, we do not report significant differences in pre-operative (*p* = 0.447), post-operative BCVA (*p* = 0.195) or logMAR gain (*p* = 0.643) between patients that were phakic (*n* = 21) or pseudophakic (*n* = 10) pre-operatively (see table, Supplementary Materials, Table S1). BCVA significantly improved in both patients that were phakic pre-operatively (from 0.47 [0.22] logMAR to 0.33 [0.16] logMAR) (*p* = 0.003) and in patients that were pseudophakic preoperatively (from 0.53 [0.22] logMAR to 0.43 [0.24] logMAR) (*p* = 0.049). Furthermore, we included pre-operative lens status as factor in our multivariate linear regression model and found that cataract surgery was not linked to significant improvement in post-operative BCVA (Table 2).

### 3.2. Pre-Operative OCT Parameters and Visual Outcomes

**Figure 3:**
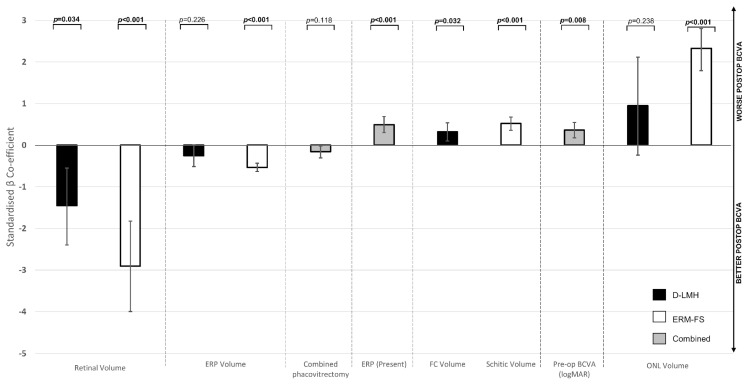
Multivariable linear regression standardized Beta coefficients and 95% confidence intervals for post-operative BCVA. This figure presents the results of a multivariable linear regression analysis examining the relationship between various predictors and post-operative BCVA. The 95% CIs provide a range of values within which we can be 95% confident that the true Beta coefficient lies. The standardized Beta coefficients represent the strength and direction of the relationship between each predictor and the post-operative BCVA, with larger absolute values indicating stronger relationships. Indeed, the table shows that retinal volume and ONL volume were the most important parameters evaluated for post-operative BCVA, as demonstrated by the higher standardized Beta coefficients, reflecting the relative importance of the independent variables for final visual outcome. The negative values of standardized Beta coefficients for pre-operative retinal volume highlight that higher retinal volumes in both D-LMH and ERM-FS subgroups are related to better post-operative BCVA (see vertical arrows on the right side of the figure). On the contrary, the positive values of standardized Beta coefficients for pre-operative ONL volume highlight that higher ONL volumes are related to worse post-operative BCVA (however, this result is statistically significant only for the ERM-FS subgroup as shown by the *p* values on the top of the figure). Legend: ONL: outer nuclear layer; ERP: epiretinal proliferation; D-LMH: degenerative lamellar macular hole; ERM-FS: epiretinal membrane foveoschisis; FC: foveal cavity; Pre-op: pre-operative. Statistical significance in bold (*p* < 0.05). The 95% CI and *p* values are based on 16,000 Wild Bootstrap.

Using the univariate tests, worse pre-operative BCVA (*p* < 0.001), presence of ERP (*p* = 0.006), and increased SV in ERM-FS (*p* = 0.008) were associated with worse functional outcomes after surgery (Table 2). No statistically significant correlation was found with post-operative BCVA for the following: sex (*p* = 0.241), age, lens status (*p* = 0.195), CRT (*p* = 0.212), MRT (*p* = 0.100), ONL thickness (*p* = 0.679), pre-operative status of EZ or ELM (respectively *p* = 0.097 and *p* = 0.447 for normal, *p* = 0.252 and *p* = 0.832 for disrupted and *p* = 0.339 and *p* = 0.141 for absent), and horizontal diameter of FC in D-LMH or foveoschisis in ERM-FS (*p* = 0.166 for maximum, *p* = 0.337 for minimum horizontal diameter). With the multivariate linear regression model following a Wild Bootstrap (16,000 resamples), presence of ERP (*p* = 0.002) and worse pre-operative BCVA (*p* = 0.008) were confirmed as significantly associated with worse final visual function. Moreover, in the subtype of ERM-FS, a lower CRV (*p* < 0.001) and ERP Volume (*p* < 0.001), a higher SV (*p* < 0.001) and foveal ONL volume (*p* < 0.001) were significantly associated with worse visual outcomes after surgery. In the D-LMH subtype, increased FC volume (*p* = 0.032) and lower CRV (*p* = 0.034) were significantly associated with worse post-operative BCVA (Table 2, Figure 3). The regression model could explain 88.8% of variability in post-operative BCVA (adjusted r^2^: 0.842). In contrast pre-operative BCVA alone could explain 44.4% of the variability in post-operative BCVA (adjusted r^2^: 0.425). Standardized Beta coefficients demonstrate the relative importance of each independent and moderator variable for post-operative BCVA (dependent variable). By reporting the standardized coefficients (Figure 3), we found that CRV in both subtypes and foveal ONL volume in ERM-FS were the most significant moderators in predicting post-operative BCVA.

A correlation matrix demonstrated that, at baseline, the increased volume of ERP strongly correlates to increased SV (*p* = 0.005), reduced CRV (*p* = 0.002) and foveal ONL volume (*p* = 0.008), lower MRT (*p* = 0.014) and ONL thickness (*p* = 0.007). Among other correlations between the predictors determined in the study, only CRV showed correlation with ONL volume (*p* < 0.001) (Supplementary Materials, Table S2). Additionally, we found an association between low pre-operative BCVA and absence of pre-operative EZ (*p* = 0.046) and presence of ERP with both poor pre- and post-operative BCVA (*p* = 0.026 and *p* = 0.006, respectively) (Supplementary Materials, Table S1).

**Figure 4:**
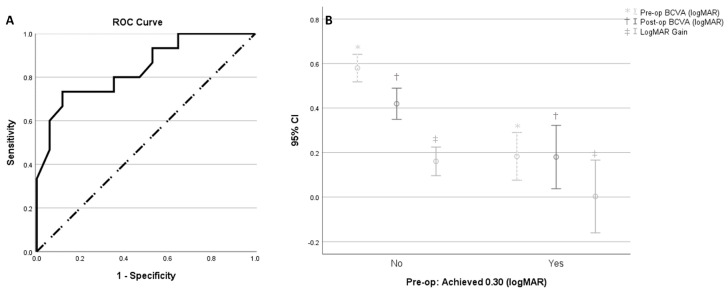
Effect of pre-operative visual acuity on visual outcomes. (**A**) Receiver Operating Characteristic (ROC) analysis using Area Under Curve (AUC) determines cut-off pre-operative BCVA of ≤0.48 logMAR as a predictor for achieving 0.30 logMAR at final follow-up (Sensitivity: 71.4%, Specificity of 88.2%, AUC 0.828 [95%CI 0.681 to 0.974], *p* = 0.002, Youden Index [J] of 0.596). (**B**) Error bar chart (95% confidence interval) of pre-operative and post-operative mean BCVA (logMAR) and logMAR gain grouped by pre-operative BCVA achieving 0.30 logMAR. Despite eyes in the worse pre-operative BCVA group having significantly larger logMAR gain, (*p* = 0.027) they also had worse post-op BCVA (*p* = 0.002).

Finally, through ROC curve and using AUC analysis, we determined a cut-off pre-operative BCVA of ≤0.48 logMAR as a predictor for achieving 0.30 logMAR at final follow up (sensitivity: 71.4%, specificity: 88.2%, AUC 0.828 [95% CI 0.681 to 0.974], *p* = 0.002, Youden Index [J]: 0.596) (Figure 4A). While significance was maintained for eyes with pre-operative BCVA > 0.30 logMAR, there was no significant change in BCVA in patients with good pre-operative BCVA (<0.30 logMAR) (*p* = 0.967). Eight (26%) of thirty-one eyes had 0.30 logMAR or better pre-operative BCVA, of which seven (88%) had maintained this post-operatively. However, among the twenty-three (84%) patients with pre-operative BCVA worse than 0.30 logMAR, eight (35%) achieved post-operative BCVA of 0.30 logMAR units or better. Despite having significantly larger logMAR gain (*p* = 0.027), eyes with worse pre-operative BCVA had worse post-operative BCVA (*p* = 0.002) (Figure 4B).

## 4. Discussion

We evaluated eyes that underwent vitrectomy with ERM/ILM peeling for LMH to investigate potential biomarkers of visual outcome. Surgical indications in eyes with LMH remain a controversial topic, mainly due to the contentious outcomes described following surgery [13,14,15]. Indeed, improvement in BCVA and/or OCT-based foveal features (such as an increase in central retinal thickness or resolution of foveal defect) have been reported after surgery for LMH [9,11,16,17,18,19,20,21]; at the same time, caution has been advised by some when choosing surgery [14,17,22,23,24]. Several studies have previously investigated the possible pre-operative predictors for surgical outcomes, but no consensus was achieved [13,14,17,22,25,26,27]. Our choice to investigate OCT volumes is based on the concept that a volumetric analysis could better represent the retinal three-dimensional structural changes in LMH.

We identified pre-operative BCVA as the main clinical parameter for final BCVA, explaining alone 44.4% of the variability in post-operative BCVA (r^2^: 0.444, adjusted r^2^: 0.425). This is consistent with previously described findings reporting a positive correlation between pre-operative BCVA and post-operative BCVA following LMH repair [10,21,25,28]. In our study, BCVA significantly improved from 0.48 (0.22) before surgery to 0.36 (0.19) logMAR after surgery. Moreover, we identified a cut off of a pre-operative BCVA better than 0.48 logMAR as a predictor factor to achieve a post-operative BCVA of 0.30 logMAR or better (with a sensitivity of 71.4% and a specificity of 88.2%). However, we also showed that visual gain was particularly significant in eyes with pre-operative BCVA worse than 0.30 logMAR. These findings mean that, on one hand, patients with LMH have the best chance of keeping driving standard vision (0.30 logMAR) if surgery is carried out before the BCVA drops to 0.48 or worse; but on the other hand, early surgery might imply small or no visual gain and patients should be informed that, in some cases, early surgery just stabilizes visual acuity.

Our study confirmed the negative association between the presence of ERP and post-surgical visual outcome (*p* = 0.006 and *p* = 0.020 for univariate and multivariate regression, respectively), as previously demonstrated in the scientific literature [13,14]. Interestingly, in the D-LMH group, increased FC volume (*p* = 0.032) and lower CRV (*p* = 0.034) showed a correlation with worse post-operative BCVA. In the ERM-FS group, lower CRV (*p* < 0.001) and ERP volume (*p* < 0.001), increased SV (*p* < 0.001) and higher foveal ONL volume (*p* < 0.001) were all significant moderators associated with worse visual outcomes after surgery in the multivariate regression. Caution should be advised to not interpret these as main effects, but as significant moderators, to avoid a Table 2 fallacy error [29]. In the scientific literature, only the study of Taşlıpınar Uzel et al. analyzed retinal volumes in the context of LMH, focusing on FC and ERP volumes in the natural history of D-LMH eyes that did not undergo surgery [6]. Similarly to previous studies [30,31,32], Taşlıpınar Uzel et al. confirmed no correlation between vision and linear OCT measurements such as CRT in D-LMH, with FC volume the only factor associated with baseline BCVA on multivariate regression [6]. CRV or ONL volume have never been investigated in the natural history, or correlated with surgical outcomes, in LMHs. To the best of our knowledge, this is the first report on pre-operative volumetric analysis of surgically treated LMHs. In the ERM-FS subtype, correlation between higher SV and worse visual outcome could be explained by the tractional forces of the ERM: larger SV might be present in more severe stages of the pathology with augmented tractional forces due to the ERM. Similarly, in D-LMH, we supposed that higher FC volumes reflect the increased degree of retinal tissue loss in the pathogenesis of the degenerative disease, indicating an advanced stage of the pathology and consequent worse visual outcomes after surgery. Moreover, smaller pre-operative CRV in our cohort (which correlates with larger SV in ERM-FS and higher FC in D-LMH) indicates a reduction in the viable retinal tissue and its volume (Figure 2), leading to poor functional outcomes post-surgery. We demonstrate that a higher foveal ONL volume is a negative moderator for post-operative BCVA in ERM-FS. We believe that this finding may appear consistent with what has been already demonstrated for idiopathic ERM [33,34]. In the view of the causative association between tractional ERM and the development of foveoschisis in ERM-FS [2], we suggest that ERM-FS may share some characteristics with eyes affected by ERM, including the association between increased ONL volume and impaired visual function. In summary, the increased schisis and higher ONL volumes point to the increased tractional effect of ERM on the retinal tissue, with a possible effect on photoreceptors, which may explain worse functional outcomes post-surgery. In the D-LMH group, lower CRV (*p* = 0.034) and higher FC volume (*p* = 0.032), both indicating a greater loss of retinal tissue, had a correlation with reduced final vision.

Vitrectomy combined with cataract surgery is a critical confounder in studies that report on visual outcomes following surgical intervention for LMH. In our cohort, we found that the combined phacovitrectomy group, relative to patients pseudophakic at baseline, gained an additional 0.03 logMAR, which was not significant. We report significant improvement in vision, whether the patient had cataract surgery, or was pseudophakic at baseline, without measured benefit to visual outcomes on the multivariate regression model by having cataract surgery. This is most likely due to our cohort of patients not having significant lenticular opacity at baseline and cataract surgery was primarily performed prophylactically with vitrectomy.

We acknowledge that this study has several limitations, including its retrospective nature, the limited number of patients and a relatively short follow-up time. Nonetheless, we conducted a precise analysis of the asymmetric retinal morphology in LMHs by using a standardized OCT protocol. Finally, this is the first study, to our knowledge, to perform a volumetric analysis on eyes that underwent PPV for LMH and we presented a well-fitting robust regression model.

## 5. Conclusions

In conclusion, our study highlights clinical and novel OCT volumetric biomarkers that correlate with post-operative BCVA following surgical intervention in LMHs. Although pre-operative BCVA was confirmed as a strong predictor of final visual outcome, the volumetric pre-operative independent variables included in our regression model offered a superior fit. We describe novel pre-operative OCT parameters significantly associated with visual outcomes following surgery such as CRV, ERP volume, SV and foveal ONL volume in ERM-FS, and CRV and FC volume in D-LMH. Further studies with larger numbers and longer post-operative follow-up can better investigate the role of volumetric analysis in the surgical treatment of lamellar macular holes.

## Figures and Tables

**Table 1:** Baseline demographics, OCT parameters and visual outcomes of eyes with LMHs, further divided in D-LMH and ERM-FS groups. The table shows the pre-operative parameters collected and the visual outcomes of the total number of eyes included in the study and of the two separated groups (ERM-FS and D-LMH). A comparison for each single parameter was performed between the D-LMH and the ERM-FS group, whose *p* values are reported in the last column of the table and statistical significance is highlighted in bold. Relative to D-LMH, ERM-FS had lower proportion of ERP (four [24%] compared to nine [64%]) (*p* = 0.033), a lower ERP volume (0.029 compared to 0.078 mm^3^) (*p* = 0.044) and higher foveal ONL average thickness and volume (113 µm compared to 90 µm, *p* < 0.001 and 0.089 mm^3^ compared to 0.07 mm^3^, *p* = 0.002). No statistically significant difference was found for the other parameters evaluated.

	Total	D-LMH	ERM-FS	*p* Value
**Total**	31	14	17	-
**Baseline Characteristics**				
Age (years)	69 (9)	65 (8)	73 (8)	0.016
Gender (% male)	10 (32%)	6 (43%)	4 (24%)	0.441
Laterality (% right)	14 (45%)	8 (57%)	6 (35%)	0.289
Phakic (% yes)	21 (68%)	12 (86%)	9 (53%)	0.068
Ellipsoid Zone				
Normal	20 (65%)	7 (50%)	13 (77%)	0.153
Disrupted	8 (26%)	6 (43%)	2 (12%)	0.097
Absent	3 (10%)	1 (7%)	2 (12%)	1.000
External Limiting Membrane				
Normal	22 (71%)	9 (64%)	13 (77%)	0.693
Disrupted	6 (19%)	3 (21%)	3 (18%)	1.000
Absent	3 (10%)	2 (14%)	1 (6%)	0.576
ERP (% yes)	13 (42%)	9 (64%)	4 (24%)	**0.033**
Pre-operative BCVA (logMAR)	0.49 (0.22)	0.50 (0.24)	0.49 (0.20)	0.897
Central Retinal Thickness	197 (82)	166 (41)	223 (99)	0.052
Minimum Retinal Thickness	155 (74)	110 (36)	191 (78)	0.001
Cavity or schisis max horizontal diameter	1292 (617)	967 (473)	1560 (603)	0.006
Cavity or schisis min horizontal diameter	428 (212)	490 (249)	377 (166)	0.139
ONL Average Thickness	102 (20)	90 (13)	113 (19)	**<0.001**
Foveal Cavity Volume	0.0716 (0.0426)	0.0716 (0.0426)	-	-
Schitic Volume	0.1057 (0.0860)	-	0.1057 (0.0860)	-
ERP Volume	0.0632 (0.0811)	0.0783 (0.0929)	0.0292 (0.0324)	**0.044**
Foveal ONL Volume	0.0803 (0.0176)	0.0700 (0.0118)	0.0888 (0.0173)	**0.002**
Central Retinal Volume	2.7945 (0.4031)	2.6509 (0.4442)	2.9127 (0.3339)	0.071
**Visual outcomes**				
Post-operative BCVA (logMAR)	0.37 (0.19)	0.42 (0.18)	0.32 (0.20)	0.184
LogMAR gain	0.12 (0.17)	0.08 (0.14)	0.16 (0.18)	0.184

Legend. ERP: epiretinal proliferation; ONL: outer nuclear layer; BCVA: best corrected visual acuity. Continuous variables are reported as mean (standard deviation, SD) and compared using independent *t*-test. Nominal variables were compared using Fisher’s exact test.

**Table 2:** Univariate and multivariate linear regression model for post-operative BCVA. On univariate tests, ERP, increased volume of schitic cavity and worse pre-operative BCVA were associated with worse post-operative BCVA. On multivariate linear regression, the presence of ERP and worse pre-operative BCVA were associated with worse visual outcomes. Interaction terms show that lower retinal volume was associated with worse visual outcomes in both subtypes. Specifically, for ERM-FS, ERP volume, increased SV and ONL volume were linked to worse post-operative BCVA. For D-LMH, we found that larger FC was associated with worse post-operative BCVA.

	Univariate		Multivariate	
Independent Variable	Post-Operative BCVA (r)	*p* Value	B Coefficient (95% CI)	*p* Value
**Pre-operative BCVA (logMAR)**	0.666	**<0.001**	**0.316 (0.155 to 0.477)**	**0.008**
Combined Phacovitrectomy	0.33 (0.16) *	0.195 *	−0.064 (−0.123 to −0.008)	0.118
Pseudophakic baseline	0.43 (0.24) *		-	-
**ERP (present)**	0.47 (0.19) *	0.006 *	**0.155 (0.082 to 0.227)**	**0.002**
**ERP (absent)**	0.29 (0.15) *		-	-
**Volume of ERP**	0.203	0.274	-	-
D-LMH	0.169	0.564	−0.793 (−1.629 to 0.013)	0.226
**ERM-FS**	0.117	0.655	**−7.116 (−8.401 to −5.765)**	**<0.001**
**Volume of Foveal Cavity (D-LMH)**	0.033	0.91	**1.335 (0.425 to 2.245)**	**0.032**
**Schitic Volume (ERM-FS)**	0.618	**0.008**	**1.197 (0.831 to 1.551)**	**<0.001**
**Retinal Volume**	−0.272	0.138	-	-
**D-LMH**	−0.141	0.63	**−0.201 (−0.332 to −0.076)**	**0.034**
**ERM-FS**	−0.284	0.27	**−0.370 (−0.509 to −0.232)**	**<0.001**
**ONL Volume**	−0.004	0.981	-	-
D-LMH	0.089	0.763	4.994 (−1.241 to 11.104)	0.236
**ERM-FS**	0.194	0.457	**9.486 (7.291 to 11.416)**	**<0.001**

Multivariate Model: R^2^ = 0.888, adjusted R^2^: 0.824. Univariate analysis Pearson correlation (* mean [standard deviation] logMAR reported, independent *t*-test). Significance defined as *p* < 0.05 and highlighted in bold. Presented 95% confidence interval and *p* values are calculated following 16,000 resampling Wild Bootstrap.

## Data Availability

Data are contained within the article and Supplementary Materials.

## Supplementary Materials

The following supporting information can be downloaded at: https://www.mdpi.com/article/10.3390/jpm14070755/s1; Figure S1: effect of pre-operative visual acuity on visual outcomes; Table S1: correlation between clinical and OCT-based parameters and pre- and post-operative visual acuity; Table S2: correlation matrix of pre- and post-operative BCVA and OCT parameters.
