# Supplementary material for: Volumetric Biomarkers of Visual Outcome after Surgical Repair in Lamellar Macular Holes
Source: J Pers Med. 2024 Jul 16;14(7):755. doi: 10.3390/jpm14070755 (PMC11278409; doi:10.3390/jpm14070755)
Supplement: Supplementary file 1 [file jpm-14-00755-s001.zip › Figure S1.pdf]

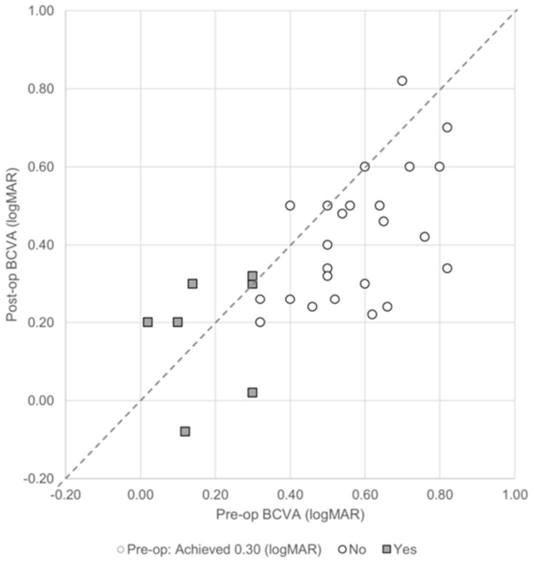

Figure S1 Effect of pre-operative visual acuity on visual outcomes

Scatter plot diagram to show the distribution of pre and post-operative best corrected visual acuity grouped by pre-operative visual acuity.
Mean pre-op BCVA improved from 0.48 (0.22) to 0.36 (0.19) ($p<0.001$) across the whole cohort. While significance was maintained for eyes with pre-op BCVA worse than pre-op BCVA≥0.30, there was no significant change in VA in patients with good pre-op BCVA (<0.30 logMAR) ($p=0.933$).
