# Supplementary material for: Volumetric Biomarkers of Visual Outcome after Surgical Repair in Lamellar Macular Holes
Source: J Pers Med. 2024 Jul 16;14(7):755. doi: 10.3390/jpm14070755 (PMC11278409; doi:10.3390/jpm14070755)
Supplement: Supplementary file 1 [file jpm-14-00755-s001.zip › Table S1.pdf]

Pre-operative Characteristics	Pre-operative BCVA (logMAR)			Post-operative BCVA (logMAR)		
	No	Yes	p Value	No	Yes	p Value
Gender: Male	0.50 (0.21)	0.47 (0.24)	0.757	0.34 (0.18)	0.42 (0.22)	0.241
Laterality: Right	0.50 (0.22)	0.48 (0.22)	0.809	0.39 (0.20)	0.34 (0.19)	0.466
Phakic	0.53 (0.22)	0.47 (0.22)	0.447	0.43 (0.24)	0.33 (0.16)	0.195
Ellipsoid Zone						
Normal	0.59 (0.23)	0.44 (0.20)	0.07	0.44 (0.21)	0.32 (0.17)	0.097
Disrupted	0.48 (0.21)	0.53 (0.25)	0.531	0.34 (0.19)	0.43 (0.19)	0.252
Absent	0.46 (0.21)	0.73 (0.08)	**0.046**	0.35 (0.18)	0.47 (0.31)	0.339
External Limiting Membrane						
Normal	0.56 (0.24)	0.46 (0.21)	0.26	0.41 (0.22)	0.35 (0.18)	0.447
Disrupted	0.49 (0.20)	0.51 (0.29)	0.839	0.37 (0.20)	0.35 (0.18)	0.832
Absent	0.47 (0.22)	0.67 (0.03)	0.142	0.35 (0.18)	0.52 (0.29)	0.141
ERP (Present)	0.42 (0.20)	0.59 (0.21)	**0.026**	0.29 (0.15)	0.47 (0.19)	**0.006**

Table S1. correlation between clinical and OCT-based parameters and pre- and post-operative visual acuity.

The table shows the correlation between specific pre-operative characteristics with baseline and final BCVA. In specific, the table shows a significant correlation between pre-operative BCVA and absence of pre-operative EZ(p=0.046) and presence of ERP correlate both with pre- and post-operative BCVA (p=0.026 and p=0.006 respectively). No significant difference was reported in pre- or post-operative BCVA or visual gain (p=0.643) between patients that were phakic or pseudophakic at baseline (significance highlighted in bolt).
