# Supplementary material for: Volumetric Biomarkers of Visual Outcome after Surgical Repair in Lamellar Macular Holes
Source: J Pers Med. 2024 Jul 16;14(7):755. doi: 10.3390/jpm14070755 (PMC11278409; doi:10.3390/jpm14070755)
Supplement: Supplementary file 1 [file jpm-14-00755-s001.zip › Table S2.pdf]

Correlation Matrix		Pre-operative BCVA (logMAR)	Post-operative BCVA (logMAR)	Minimal Retinal Thickness	Central Retinal Thickness	ONL Average Thickness	Cavity or schisis max horizontal diameter	Cavity or schisis min horizontal diameter	Schitic Volume	Volume of Foveal Cavity	Volume of ERP	Foveal ONL Volume	Central Retinal Volume
Pre-operative BCVA (logMAR)	Pearson Correlation	1	0.666**	-0.061	-0.02	-0.036	0.183	0.069	0.289	0.078	0.243	0.023	-0.018
	Sig. (2-tailed)		<0.001	0.745	0.916	0.846	0.324	0.711	0.261	0.791	0.188	0.904	0.922
Post-operative BCVA (logMAR)	Pearson Correlation	0.666**	1	-0.301	-0.23	-0.077	0.255	0.178	0.618**	0.033	0.203	-0.004	-0.272
	Sig. (2-tailed)	<0.001		0.100	0.212	0.679	0.166	0.337	0.008	0.91	0.274	0.981	0.138
Minimal Retinal Thickness	Pearson Correlation	-0.061	-0.301	1	0.720**	0.458**	0.095	-0.202	-0.45	0.084	-0.438*	0.479**	0.482**
	Sig. (2-tailed)	0.745	0.100		<0.001	0.01	0.610	0.275	0.07	0.776	0.014	0.006	0.006
Central Retinal Thickness	Pearson Correlation	-0.02	-0.23	0.720**	1	0.449*	0.103	-0.148	-0.366	0.313	-0.02	0.449*	0.366*
	Sig. (2-tailed)	0.916	0.212	<0.001		0.011	0.581	0.427	0.149	0.275	0.913	0.011	0.043
ONL Average Thickness	Pearson Correlation	-0.036	-0.077	0.458**	0.449*	1	0.041	0.444*	-0.314	-0.026	-0.473**	0.986**	0.711**
	Sig. (2-tailed)	0.846	0.679	0.01	0.011		0.826	0.012	0.219	0.929	0.007	<0.001	<0.001
Cavity or schisis max horizontal diameter	Pearson Correlation	0.183	0.255	0.095	0.103	0.041	1	0.272	0.742**	0.839**	-0.006	0.039	-0.187
	Sig. (2-tailed)	0.324	0.166	0.610	0.581	0.826		0.139	<0.001	<0.001	0.975	0.836	0.313
Cavity or schisis min horizontal diameter	Pearson Correlation	0.069	0.178	-0.202	-0.148	-0.444*	0.272	1	0.071	0.589*	0.278	-0.437*	-0.421*
	Sig. (2-tailed)	0.711	0.337	0.275	0.427	0.012	0.139		0.788	0.027	0.129	0.014	0.018
Schitic Volume	Pearson Correlation	0.289	0.618**	-0.450	-0.366	-0.314	0.742**	0.071	1	-	0.648**	-0.271	-0.480
	Sig. (2-tailed)	0.261	0.008	0.070	0.149	0.219	<0.001	0.788		-	0.005	0.293	0.051
Volume of Foveal Cavity	Pearson Correlation	0.078	0.033	0.084	0.313	-0.026	0.839**	0.589*	-	1	0.155	-0.077	0.06
	Sig. (2-tailed)	0.791	0.91	0.776	0.275	0.929	<0.001	0.027	-		0.596	0.793	0.837
Volume of ERP	Pearson Correlation	0.243	0.203	-0.438*	-0.02	-0.473**	-0.006	0.278	0.648**	0.155	1	-0.468**	-0.543**
	Sig. (2-tailed)	0.188	0.274	0.014	0.913	0.007	0.975	0.129	0.005	0.596		0.008	0.002
Foveal ONL Volume	Pearson Correlation	0.023	-0.004	0.479**	0.449*	0.986**	0.039	0.437*	-0.271	-0.077	-0.468**	1	0.706**
	Sig. (2-tailed)	0.904	0.981	0.006	0.011	<0.001	0.836	0.014	0.293	0.793	0.008		<0.001
Central Retinal Volume	Pearson Correlation	-0.018	-0.272	0.482**	0.366*	0.711**	-0.187	-0.421*	-0.48	0.06	-0.543**	0.706**	1
	Sig. (2-tailed)	0.922	0.138	0.006	0.043	<0.001	0.313	0.018	0.051	0.837	0.002	<0.001	

**Correlation is significant at the 0.01 level (2-tailed)0.

*Correlation is significant at the 0.05 level (2-tailed)0.

Table S2. Correlation matrix of pre- and post-operative BCVA and OCT parameters.

The table shows a correlation matrix for different parameters evaluated. Interestingly, in the table, a correlation matrix is reported demonstrating that, at baseline, increased volume of ERP strongly correlate to increased SV (p=0.005), reduced CRV (p=0.002) and foveal ONL volume (p=0.008), lower MRT (p=0.014) and ONL thickness (p=0.007).
